# Acute and long‐term efficacy of ablation index‐guided higher power shorter duration ablation in patients with atrial fibrillation: A prospective registry

**DOI:** 10.1002/joa3.12605

**Published:** 2021-07-21

**Authors:** So‐Ryoung Lee, Hyoung‐Seob Park, Eue‐Keun Choi, Euijae Lee, Seil Oh

**Affiliations:** ^1^ Department of Internal Medicine Seoul National University Hospital Seoul Republic of Korea; ^2^ Division of Cardiology Department of Internal Medicine Dongsan Medical Center Keimyung University Daegu Republic of Korea

**Keywords:** ablation index, atrial fibrillation, high power ablation, pulmonary vein isolation

## Abstract

**Background:**

Theoretically, targeting the same ablation index (AI) using higher power may achieve the same lesion size with a shorter ablation time. We evaluated the acute and long‐term efficacy of higher‐powered ablation guided by ablation index (HPAI) compared with conventional‐powered ablation guided by AI (CPAI) for pulmonary vein isolation (PVI) in patients with atrial fibrillation (AF).

**Methods:**

Drug refractory symptomatic AF patients who had been ablated with 40 W on the anterior/roof segments and 30 W on the posterior/inferior/carina segments were enrolled (HPAI group). We compared the HPAI group with the CPAI group who were ablated with 30 W on the anterior/roof segments and 25 W on the posterior/inferior/carina segments. The same AI was targeted (≥450 on the anterior/roof segments and ≥350 on the posterior/inferior/carina segments). We compared ablation time, acute pulmonary vein reconnection (PVR) and 1‐year AF recurrence between the two groups.

**Results:**

A total of 118 patients were included (86 in the HPAI group and 32 in the CPAI group, paroxysmal AF, 73%). There was no significant difference in the acute PVR rate between the HPAI and the CPAI groups (3.7% vs. 4.2%, *P* = .580) with a 41% reduction in ablation time for PVI (38.7 ± 8.3 vs. 65.8 ± 13.7 minutes, *P* < .001). The 1‐year AF recurrence rate was not significantly different between HPAI and CPAI groups (12.8% vs. 21.9%, Log‐rank *P* = .242). There were no major complications in either group.

**Conclusions:**

Increased power during AF ablation, using the same AI targets, reduced the procedure and ablation times, and showed a comparable acute and long‐term outcome without compromising safety.

**Clinical Trial Registration:**

https://www.clinicaltrials.gov. Unique identifier: NCT 04379557.

## Graphical abstract

There are limited data regarding the acute and long‐term efficacy and safety of applying higher power than conventional power targeting optimal ablation index (AI) value in our daily pulmonary vein isolation (PVI) procedure in patients with atrial fibrillation. In this study, we found that higher‐than‐conventional radiofrequency power PVI guided by optimal target AI showed comparable acute and long‐term efficacy of PVI with reducing the procedure and ablation times without increasing the risk of complication. This study has shown that higher power strategy could be successfully implemented on the basis of conventional power AI guided PVI.

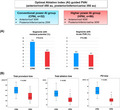

## 1 INTRODUCTION

Catheter ablation of atrial fibrillation (AF) to achieve pulmonary vein isolation (PVI) is an effective treatment for rhythm management.^1^, ^2^ Recently, there have been several technological advances in catheter ablation of AF that have helped to achieve a more effective and safer PVI.^3^, ^4^, ^5^ The ablation index (AI) is a recently developed weighted formula, which includes contact force (CF), ablation time, and radiofrequency (RF) power that has a good correlation with lesion formation.^5^, ^6^ After the introduction of AI in clinical practice, several studies reported the efficacy and safety of AI‐guided PVI.^7^, ^8^, ^9^, ^10^ Previously, we reported that optimal AI targeted PVI (anterior/roof segments, AI 450 and posterior/inferior/carina segments, AI 350), the so‐called “OPTIMUM” protocol, showed an improved acute outcome of PVI than conventional CF‐guided strategy.^11^

A recently developed ablation catheter with surround flow (SF) and 56 irrigation holes allows safe ablation at higher RF power and may lead to rapid and effective lesion formation.^12^, ^13^, ^14^ Theoretically, targeting the same AI using higher power may achieve the same quality and size of lesion with shorter ablation times. Several studies have recently reported that PVI using higher power reduced procedure time with comparable efficacy.^15^, ^16^, ^17^, ^18^, ^19^ However, these studies applied different target AIs and different ranges of “high power,” and still, there is concern about the risk of complications, such as steam pop in a higher‐power ablation.^15^, ^16^, ^17^, ^18^, ^19^ There are limited data regarding the acute and long‐term efficacy and safety of applying higher power than conventional power targeting optimal AI value in our daily routine PVI procedure.

In this study, we aimed to evaluate how to apply a higher power in PVI guided by optimal target AI and compare the ablation time, acute and long‐term efficacy and safety between higher‐powered ablation and conventional‐powered ablation in patients with AF.

## 2 METHODS

### 2.1 Study design and study population

From July 2018 to November 2019, 86 consecutive patients with drug‐refractory symptomatic AF underwent optimal AI‐guided PVI with higher RF power (higher‐powered AI‐guided ablation, HPAI group). The results of the HPAI group were compared with those of the 32 patients enrolled in the “OPTIMUM” phase 2 study,^11^ undergoing optimal AI‐guided PVI with conventional RF power (conventional‐powered AI‐guided ablation, CPAI group). We planned to compare the HPAI group that prospectively collected with the CPAI group that pre‐registered cases to enroll the total study population more easily and efficiently as mentioned and reduce the total study duration. Immediately after the completion of the OPTIMUM study (the CPAI group),^11^ the LESS study (the HPAI group) was conducted, so there was no significant change in treatment practice, operator proficiency, and the device. Patients younger than 20 years or older than 80 years, with left atrial (LA) diameter >50 mm, and severe left ventricular systolic dysfunction (left ventricle ejection fraction <35%) were excluded. All procedures were performed by experienced operators in two tertiary centers. The study was approved by the Institutional Review Board of each center. The study was conducted according to the Declaration of Helsinki. All patients provided written informed consent before undergoing the AF ablation procedure.

### 2.2 Protocol for the PVI procedure and outcome measurement

The PVI was performed under deep conscious sedation. After 1 or 2 transseptal punctures (SL1; St. Jude Medical), a Pentaray catheter (Biosense Webster Inc,) and an open‐tip irrigated CF‐sensing RF catheter (Thermocool SmartTouch SF catheter, Biosense Webster Inc) were positioned in the LA. Three‐dimensional electroanatomical map of the LA and PVs was created (CARTO 3 system, Biosense Webster Inc). A point‐by‐point circumferential ablation was performed for PVI. For the HPAI group, RF energy was delivered in a power‐controlled mode with 40 W (irrigation flow up to 15 mL/min) at the anterior/roof segments and 30 W (irrigation flow up to 8 mL/min) at the posterior/inferior/carina segments (Figure S1). For the CPAI group, RF energy was delivered in a temperature‐controlled mode with 30‐35 W (irrigation flow up to 30 mL/min) at the anterior/roof segments and 25‐30 W (irrigation flow up to 17 mL/min) at the posterior/inferior/carina segments using an open‐tip irrigated CF‐sensing RF catheter (Thermocool SmartTouch catheter; Biosense Webster Inc).^11^ In both the HPAI and CPAI groups, an RF energy of 25 W was applied for 15 seconds at the inferior/posterior segments of the left inferior PV (LIPV) and posterior segments of the left superior PV (LSPV) near the esophagus to avoid esophageal injury. Esophageal temperature was monitored in all study patients. When the esophageal temperature increased to more than 38℃, we stopped the RF energy delivery and moved to other sites.

**FIGURE 1:**
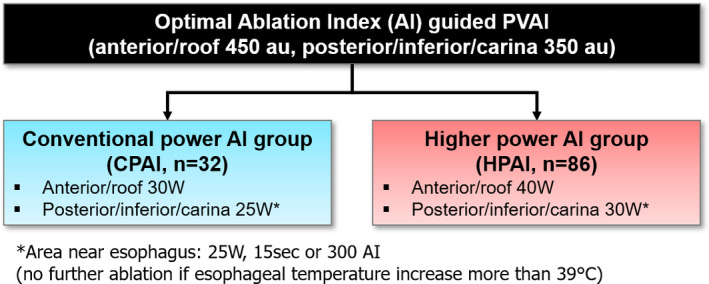
Study design. AI, ablation index; PVAI, pulmonary vein antral isolation

The target AI was based on the OPTIMUM protocol.^11^ Briefly, the OPTIMUM protocol was a point‐by‐point RF ablation for PVI with contiguous lesions. RF energy was delivered until an AI of ≥450 was attained at the anterior/roof segments, and an AI of ≥350 was attained at the posterior/inferior/carina segments. The target CF was between 5 and 20 g. Visitag (Biosense Webster Inc) was used with a predefined set of catheter stability (maximum range 2.5 mm for a minimum time of 5 seconds) and a minimum CF of 5 g with force over time 25% (3 g at the left superior and inferior ridges). Each Visitag annotation point was presented according to the AI as a lesion tag size of 2 mm (radius 2 mm ball), and the maximal interlesion distance between neighboring lesion was ≤4 mm. If the catheter dislocated before reaching the target AI, a new RF ablation was applied to reach the AI target. The study flow is summarized in Figure 1.

After the first pass ablation of all PVs, residual potential (RP) was assessed as the remaining PV potential. When the PV was not isolated after the first encirclement, additional touch‐up ablation was delivered. After a 20‐minute observation of PVI achievement, spontaneous early reconnection (ER) of PV was evaluated. Acute PVR was defined as the presence of ER. In the case of the presence of acute PVR, additional touch‐up ablation was performed to achieve PVI. Additional linear ablation or ablation for non‐PV trigger foci was performed according to the discretion of the operator. If AF persisted after ablation, internal or external electrical cardioversion was performed.

### 2.3 Demographic, procedural, and ablation lesion data analysis

Patient data on age, sex, comorbidities, CHA_2_DS_2_‐VASc score, and echocardiographic parameters were collected. Covariates, including the ablation procedure (PVI only or whether performing additional ablation other than PVI), total procedure time, total ablation time, ablation time for PVI, and fluoroscopy time, were analyzed. Segments with RP and acute PVR were analyzed by predefined 14 PV segments (Figure S1). The VisiTag data from each ablation point during PVI were analyzed. Mean CF, power, impedance drop, force‐time integral (FTI), AI, mean, and total ablation duration at each point was analyzed for each segment.

### 2.4 Acute and long‐term efficacy outcomes

We compared total procedure time, total ablation time, ablation time for PVI, and fluoroscopy time between the HPAI and CPAI groups. For assessment of acute efficacy, the rate of RP and acute PVR among PV segments were compared between the two groups.

After the index procedure, follow‐up visits were arranged after 2 weeks and 3, 6, 9, and 12‐month. A 12‐lead electrocardiogram was performed at each follow‐up visit, and a 24‐hour Holter monitoring was performed at 3‐ and 12‐month visit. The long‐term efficacy endpoint was defined as the recurrence of atrial tachycardia (AT) or AF evaluated by documented any AT or AF lasting longer than 30 seconds within 1‐year follow‐up after the 3‐month blanking period after the index procedure.^1^

### 2.5 Safety outcomes

Procedure‐related complications were defined as safety outcomes, including steam pop, cardiac tamponade, atrial‐esophageal fistula, stroke, transient ischemic attacks, and death within 4‐weeks and 1‐year after the index procedure.

### 2.6 Preclinical study: comparison of the lesion size between 40W and 30W RF ablation at the same target AI of 450 in canine models

To provide complementary information on the ablation parameters and the lesion sizes between 40 W and 30 W RF ablation at the target AI of 450, we conducted a preclinical in vivo experiment using four mongrel dogs (32 ± 6.8 kg, males). General anesthesia was induced using intravenous tiletamine/zolazepam (Zoletil^®^, 5 mg/kg; Virbac S/A) and maintained using isoflurane gas (1%–2% oxygen). The animals were intubated and mechanically ventilated. Under sterile conditions, an open‐tip irrigated CF‐sensing catheter (Thermocool SmartTouch SF catheter, Biosense Webster Inc) was positioned in the right atrium (RA) and right ventricle (RV). Ablation was performed with the target AI of 450 (CF 10‐15 g) at the RA and RV using 40 W and 30 W, respectively. The ablation parameters, including CF, ablation time, RF power, baseline impedance, impedance drop, FTI, and AI were measured. The presence of steam pop at each ablation point was recorded. After ablation, the lesion size and depth were measured at each point. The protocol for this study was approved by the Institutional Animal Care and Use Committee of the Seoul National University Hospital (IACUC 18‐0076‐S1A0). The protocol conforms to the best practices, as defined by the Guide for the Care and Use of Laboratory Animals.

### 2.7 Statistical analysis

Categorical variables are presented as numbers and percentages, and continuous variables with normal distribution are presented as mean ± SD. The chi‐square test was performed to compare categorical variables. Continuous variables were compared with the Student's *t* test. Continuous variables with non‐normal distribution are presented as the median and interquartile range (IQR) and compared by Wilcoxon test or the Kruskal‐Wallis test. The AT/AF free survival at 1‐year between the two groups was analyzed using Kaplan‐Meier curves and log‐rank test. All tests were 2‐sided, and a *P* < .05 was considered statistically significant. All statistical analyses were performed using SPSS 25.0 (IBM Corp.).

## 3 RESULTS

### 3.1 Baseline characteristics

A total of 86 patients (mean age, 59.5 ± 9.0 years, median CHA_2_DS_2_‐VASc score, 1 [IQR 1‐2], and 77% of paroxysmal AF) were consecutively enrolled in the HPAI group. There were no significant differences between the two groups in their baseline characteristics (Table 1 and Table S1).

**TABLE 1:** Baseline characteristics and procedural data of study groups

	HPAI group (n = 86)	CPAI group (n = 32)	*P*‐value
Age	59.5 ± 9.0	59.9 ± 9.1	.830
Male	66 (76.7%)	25 (78.1%)	.874
Body mass index (kg/m^2^)	24.9 ± 3.0	24.8 ± 2.8	.753
Paroxysmal AF	62 (72.1%)	24 (75.0%)	.752
Hypertension	36 (41.9%)	10 (31.2%)	.293
Diabetes mellitus	17 (19.8%)	6 (18.8%)	.901
Heart failure	1 (1.2%)	1 (3.1%)	.463
History of stroke/TIA	5 (5.8%)	3 (9.4%)	.494
Vascular disease*	1 (1.2%)	0 (0%)	.540
CHA_2_DS_2_‐VASc score	1 (1‐2)	1 (0‐2)	.680
LVEF (%)	59.9 ± 4.8	59.2 ± 5.1	.445
LA AP diameter (mm)	42.6 ± 4.5	41.2 ± 5.4	.203
LA volume index (mL/m^2^)	46.7 ± 13.6	53.7 ± 20.3	.381
Procedure characteristics			
PVI only	32 (37.2%)	12 (37.5%)	.977
Additional ablation	54 (62.8%)	20 (62.5%)	
PVI time (min)	38.7 ± 8.3	65.8 ± 13.7	<.001
Total ablation time (min)	48.1 ± 13.8	77.7 ± 20.1	<.001
Total procedure time (min)	139.9 ± 37.9	199.1 ± 41.6	<.001
Fluoroscopic time (min)	14.3 ± 5.9	19.8 ± 7.3	<.001

Abbreviations: AF, atrial fibrillation; AI, ablation index; CPAI, conventional‐powered AI‐guided PVI; HPAI, higher‐powered AI‐guided PVI; LA AP, left atrium anteroposterior; PVI, pulmonary vein isolation; TIA, transient ischemic attack.

* Vascular disease includes prior myocardial infarction or presence of peripheral artery disease.

### 3.2 Procedure and ablation times in HPAI and CPAI groups

**FIGURE 2:**
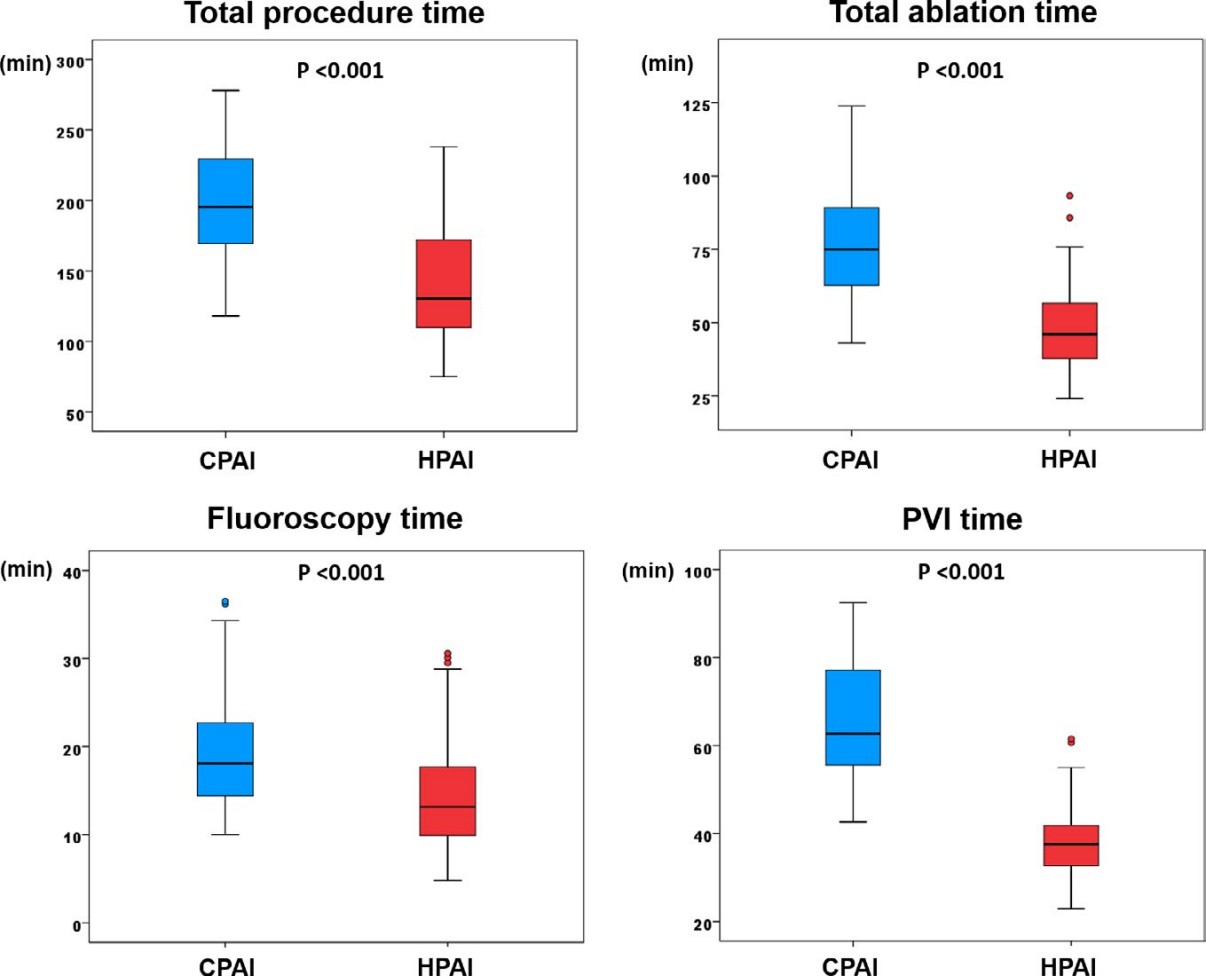
Comparison of total procedure time, fluoroscopy time, total ablation time, and ablation time for PVI between HPAI and CPAI groups. AI, ablation index; CPAI, conventional‐powered AI‐guided PVI; HPAI, higher‐powered AI‐guided PVI; PVI, pulmonary vein isolation

The HPAI group showed a shorter PVI time than the CPAI group (Table 1 and Figure 2). The shorter PVI time resulted in the reduction of total procedure time, total ablation time, and fluoroscopy time in the HPAI group compared with the CPAI group (Table 1 and Figure 2). The mean PVI time was reduced by 41%, and the mean total procedure time reduced by 30% in the HPAI group compared with the CPAI group.

### 3.3 Ablation parameters in HPAI and CPAI groups

Compared with the CPAI group, there were fewer RF applications for PVI and ablation points by each segment in the HPAI group (Table 2). In the HPAI group, the mean ablation time for each ablation was shorter and the total ablation time for each segment was shorter, whereas the mean AI was not significantly different between the two groups (Table 2, Table S2, and Figure S2). In the HPAI group, the mean power was higher, whereas CF, FTI, and impedance drop were lower than in the CPAI group. These results were consistently observed in anterior/roof segments and posterior/inferior/carina segments (Table S2).

**TABLE 2:** Ablation parameters of HPAI and CPAI groups

	HPAI group	CPAI group	*P*‐value
Total	1204 segments	448 segments	
Total number of ablation points for PVI	92.6 ± 17.7	111.7 ± 22.4	<.001
Number of ablation points by each segment	6.6 ± 3.8	8.0 ± 5.2	<.001
Total ablation time for segment (s)	134.4 ± 82.9	235.6 ± 180.0	<.001
Mean ablation time by each ablation point (s)	20.7 ± 6.5	28.4 ± 9.1	<.001
Mean power (W)	34.5 ± 5.8	28.1 ± 3.4	<.001
Mean contact force (g)	9.0 ± 2.6	10.2 ± 3.2	<.001
Mean FTI (g × s)	174.5 ± 54.5	274.6 ± 103.7	<0.001
Mean AI	390.1 ± 83.6	394.0 ± 65.5	.372
Mean impedance drop (μ)	8.7 ± 3.3	11.7 ± 4.4	<.001

Abbreviations: AI, ablation index; CPAI, conventional‐powered AI guided ablation; FTI, force‐time integral; HPAI, higher‐powered AI guided ablation; LIPV, left inferior pulmonary vein.

### 3.4 Comparison of acute and long‐term efficacy outcomes between HPAI and CPAI groups

**FIGURE 3:**
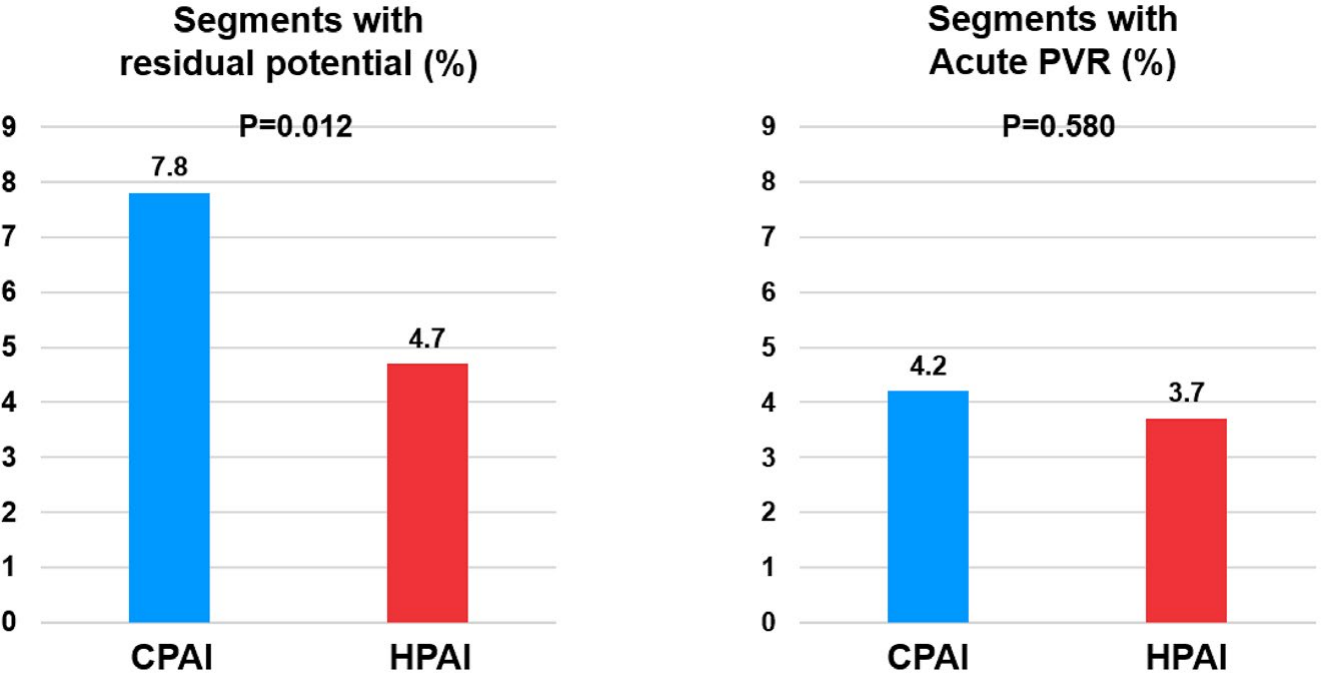
Comparison of segments with residual potential and early reconnection after PVI between HPAI and CPAI groups. AI, ablation index; CPAI, conventional‐powered AI‐guided PVI; HPAI, higher‐powered AI‐guided PVI; PVI, pulmonary vein isolation; PVR, pulmonary vein reconnection

Figure 3 shows the rates of segments with RP and acute PVR in the HPAI and CPAI groups. The HPAI group showed a lower RP rate than the CPAI group after first pass PVI (4.7% vs. 7.8%, *P* = .012). The RP and ER rates of each segment in two study groups are presented in Figure S3. The rate of acute PVR was similar between the HPAI and the CPAI group (3.7% vs. 4.2%, *P* = .580). The distributions of RP and ER by predefined PV segments are presented in Figure S3.

**FIGURE 4:**
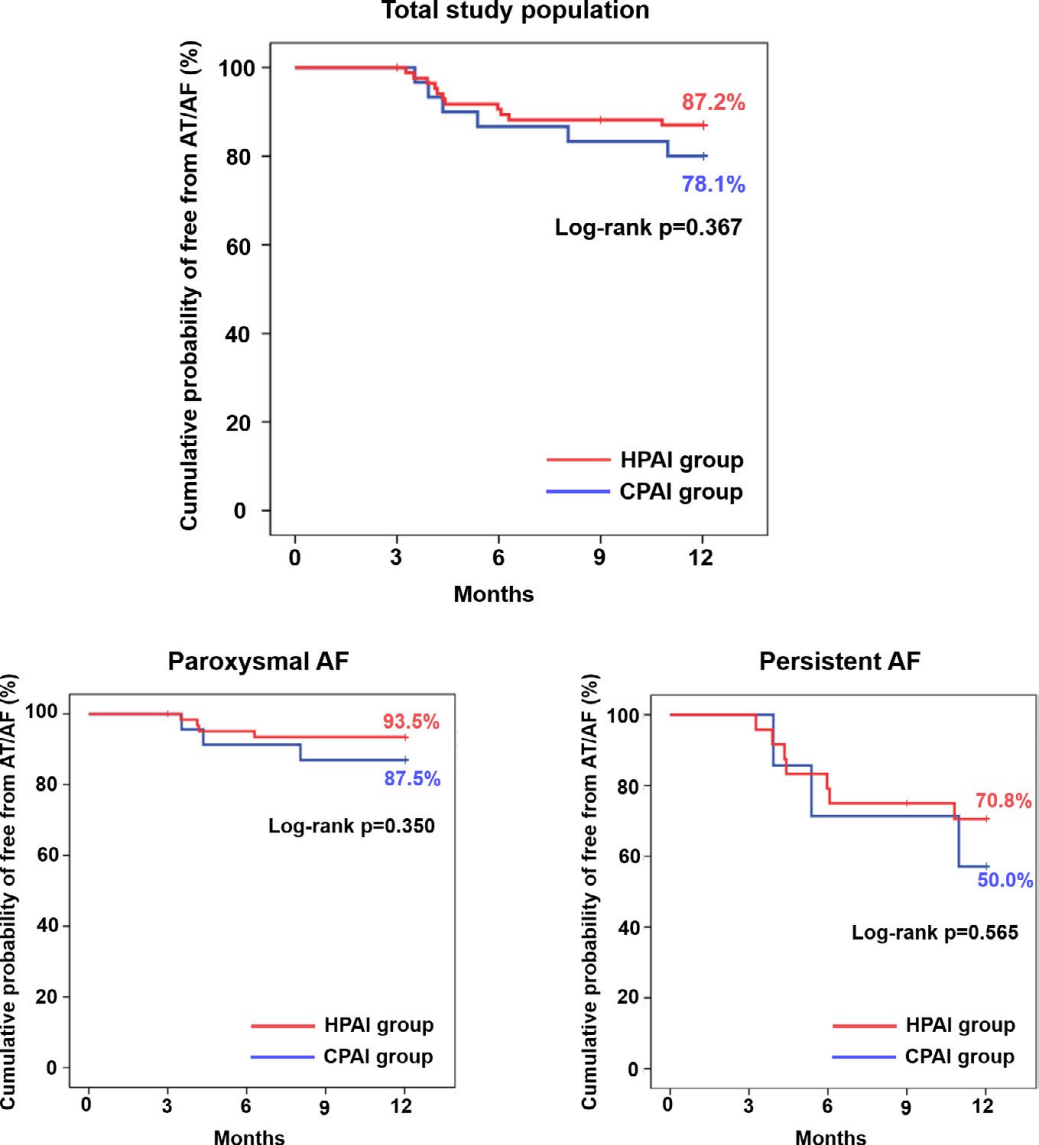
Kaplan‐Meier curve of freedom from atrial tachyarrhythmia recurrence between HPAI and CPAI groups. AF, atrial fibrillation; AT, atrial tachycardia; CPAI, conventional‐powered AI‐guided PVI; HPAI, higher‐powered AI guided PVI; PVI, pulmonary vein isolation

In the period from the end of the blanking period to the end of 12‐month follow‐up, AT/AF recurrence was documented in 12.8% of the HPAI group and 21.9% of the CPAI group. Figure 4 shows Kaplan‐Meier curves of AT/AF free survival for the two groups. There was no significant difference between the two groups (log‐rank *P* = .367). Patients with paroxysmal AF showed a higher 1‐year AF‐free survival rate than persistent AF (Figure 4). In both paroxysmal AF and persistent AF, 1‐year AT/AF survival was not different between HPAI and CPAI groups.

### 3.5 Safety outcomes

Procedural complications had not occurred within 4‐weeks and 1‐year of the procedure in the HPAI and CPAI groups.

### 3.6 Preclinical study: lesion size at target AI of 450 for 40 W and 30 W

We performed a preclinical study to compare the lesion size and ablation parameters between 40 W and 30 W deliveries with a target AI of 450 (Table S3, Figures S4 and S5). In the RA, all ablation points were transmural lesions with a mean maximum lesion diameter of 7.6 ± 0.8 mm at 40 W and 7.8 ± 1.8 mm at 30 W (*P* = .761). There was 1 steam pop in 29 lesions at 40 W (3.4%) and 1 steam pop in 31 lesions at 30 W (3.2%) (*P* = .962). In the RV, there were no significant differences in lesion size between 40 W and 30 W. Mean lesion size of the 40 W ablation was 7.7 ± 1.4, 8.3 ± 2.1, and 6.0 ± 1.4 mm, and the mean lesion size of the 30 W ablation was 8.3 ± 1.5, 9.1 ± 2.3, and 5.9 ± 1.9 mm for surface lesion width, maximum lesion width, and lesion depth, respectively (*P*‐values, .135, .273, and .841, respectively). With similar lesion size, the 40 W ablation exhibited a shorter mean ablation duration than the 30 W ablation (19.8 ± 2.4 vs. 31.1 ± 2.9 seconds, *P* < .001).

## 4 DISCUSSION

**FIGURE 5:**
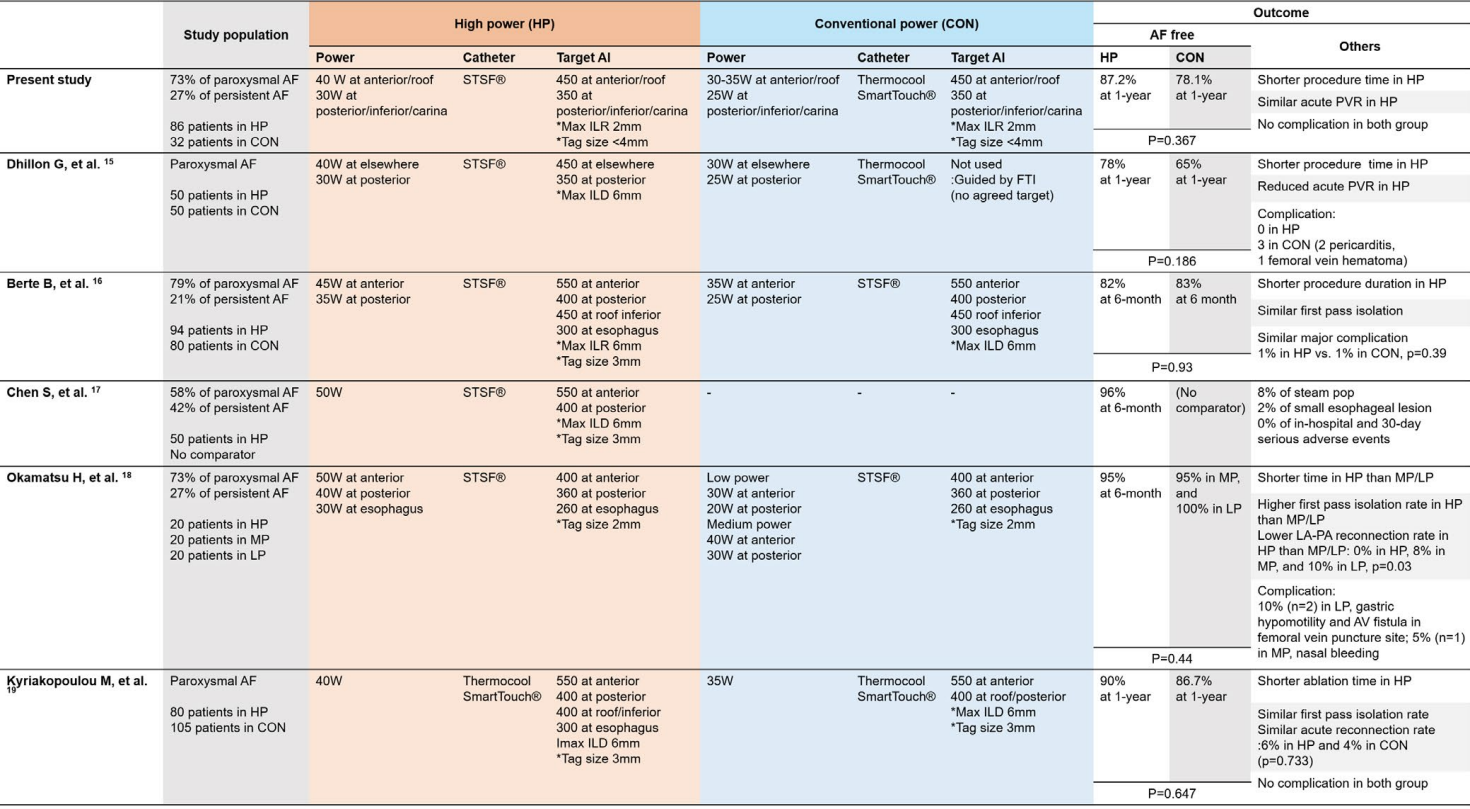
Summary of previous studies applying high RF power during PVI. AF, atrial fibrillation; AI, ablation index; CON, conventional power; HP, high power; ILD, interlesion distance; LP, low power; MP, medium power; PVI, pulmonary vein reconnection; PVR, pulmonary vein reconnection; RF, radiofrequency; STSF, SmartTouchSurroundFlow^®^

In this study, we found that higher‐than‐conventional RF power PVI guided by optimal target AI showed comparable acute and long‐term efficacy of PVI with reducing the procedure and ablation times without increasing the risk of complication. Although there have been similar studies that reported the efficacy and safety of high‐power RF ablation for patients with AF,^15^, ^16^, ^17^, ^18^, ^19^ our study investigated the efficacy and safety of “higher‐power” in the RF ablation for PVI using AI compared to the usual practice (conventional powered RF ablation) guided ablation. As mentioned in Figure 5, recent studies of high power ablation used different ablation target (eg different target AI) and compared with different comparator group for each study. In this study, we used target AI of the OPTIMUM protocol and implemented moderately higher power, 40 W at anterior/roof and 30 W at posterior/inferior/carina segments. We had demonstrated higher power strategy could be successfully implemented on the basis of conventional power AI‐guided PVI.

Using 50 W with a short duration is a new strategy for PVI to reduce collateral damage.^20^ In a previous animal study, high power, short duration atrial ablation was found to be as safe and effective as conventional ablation: 50 and 60 W ablations for 5 seconds achieved transmurality and had fewer complications compared with conventional ablation (defined as 40 W for 30 seconds).^21^ Several clinical studies have shown that high power, short duration ablation was safe, reduced total procedure time, and achieved comparable long‐term efficacy in AF recurrence.^22^, ^23^, ^24^, ^25^ In recently published data, high power short duration ablation using 50 W/8‐10 seconds in anterior and 50 W/6‐8 seconds in posterior showed similar 1‐year AF‐free survival rate and shorter procedure times compared to conventional power FTI‐guided ablation in PVI.^26^ This recent study did not apply real‐time AI or LSI estimation during ablation. However, applying 50 W in 5 seconds showed variable FTI or lesion index (LSI) values according to CF: mean FTI varied from 47 to 376 g*seconds and mean LSI varied from 4.1 to 7.6.^25^ Also, high power short duration ablation showed a higher propensity for reconnection of the right PV carina compared to conventional power ablation.^26^

After introducing values expecting the lesion size, such as AI, high power ablation was interpreted in the context of the same AI in several previous studies, as summarized in Figure 5.^15^, ^16^, ^17^, ^18^, ^19^ Rather than universal use of “5 seconds” as short duration, different ablation times derived from the formula were applied.^4^, ^15^, ^16^, ^17^, ^18^, ^19^ Although these studies shared the same hypothesis and results: by targeting the same AI with a higher power, they achieved comparable efficacy and safety with reduction of procedure and ablation times, the detailed ablation strategies were varied. Previous studies based on the “CLOSE protocol” used a higher target AI than our study: AI 550 at anterior and 400 at posterior.^16^, ^17^, ^19^ In addition, the definition of high RF power varied in these studies: variously, anterior 40, 45, and 50 W.^16^, ^17^, ^19^ In a study using 50 W with an SF catheter (single‐arm study without a comparator), there were steam pop events in 8% of the total.^17^ Okumura et al applied three ranges of RF power: low (30 W at anterior and 20 W at posterior), medium (40 W at anterior and 30 W at posterior), and high (50 W at anterior and 40 W at posterior) with a fixed target AI 400 at anterior, 300 at posterior, and 260 at the esophagus area.^18^ Dillon et al used the same RF power and the same target AI with the OPTIMUM protocol for the high power group, but did not apply AI‐guided ablation for the conventional power group.^15^ Few of the existing studies have been conducted with randomized controlled trial designs. In a recent study, different power (30 W for 40 seconds, 40 W for 20 seconds, and 50 W for 10 seconds) were randomly assigned at anterior, and fixed power (25‐30 W) was applied at posterior wall.^27^ Although PVI was not guided by AI, mean AI of three groups was not different (mean AI 436‐453). PVI with high power (50 W) short duration was effective and safe with shortened procedure time compared to conventional power PVI.^27^ In one recent study, 1.3% of esophageal erythema, 1.3% of esophageal erosion, and 15.7% of gastroparesis were reported after PVI with 50 W for 10 seconds at anterior and 50 W for 6 seconds at posterior wall.^28^ Although several studies applied 50W as a high power PVI, it can still be questionable about safety, especially at the posterior wall. Our study demonstrated the comparable efficacy and safety of higher power ablation (40 W at anterior and 30 W at posterior) compared to conventional power ablation (30 W at anterior and 25 W at posterior) using the same targeted AI (450 at anterior and 350 at posterior) in both groups. The major benefits of higher power ablation were reductions in procedure and ablation times. Higher power with the same AI naturally reduced ablation time for each ablation point compared with conventional power. Moreover, the HPAI group showed fewer segments with RP and improved first‐pass isolation rates compared with the CPAI group, whereas no complications were reported in the HPAI group. This might result in an overall reduction of total procedure time, total ablation time, and ablation time for PVI without compromising safety. In a recent updated meta‐analysis, although target power and the study protocol slightly varied, high power short duration PVI showed lower acute PVR and higher freedom from atrial arrhythmia with shorter RF ablation time without any significant differences in total complications.^29^ Namely, the efficacy and safety of higher power shorter duration PVI strategy compared to conventional strategy consistently observed in a meta‐analysis including a large number of patients (2357 in high power short duration ablation and 1361 in conventional ablation).^29^ In the context of our study results, operators can apply a high power strategy during AF ablation using target AI on the basis of conventional power PVI.

### 4.1 Study limitations

This study has several limitations. First, this is a prospective registry study when compared with a previous study group; we sequentially enrolled patients in two study groups. Therefore, the two groups were taken from two different prospective registries instead of one randomized prospective study. Although baseline characteristics of HPAI and CPAI groups were not significantly different, the results should be carefully interpreted with understanding the inherent limitation of the study design. Second, ablation was performed with a different type of catheter in the two groups (Thermocool SmartTouch catheter in the CPAI group vs. Thermocool SmartTouch SF catheter in the HPAI group). In previous studies, the SF catheter was more frequently used for high‐power PVI groups.^15^, ^16^, ^17^, ^19^ Results from a recent study, using the same catheter (Thermocool SmartTouch catheter) in both groups are consistent with ours: the high power group had reduced procedure and ablation times without any differences in the efficacy and safety of the PVI procedure.^20^ Third, the main purpose of this study was to compare the different power strategies for PVI. However, a substantial portion of patients received additional ablation other than PVI during the procedure (Table S1). To exclude the impact of additional ablation other than PVI, we enrolled a similar number of patients with persistent AF in each group. Also, we provided PVI time and acute PVR for independent interpretation for PVI. Fourth, although we believed the power difference in the same AI target was the main cause of the difference of PVI time, other factors might affect the total number of RF applications for PVI and the ablation time for PVI. The left atrium size was slightly larger in CPAI group than the HPAI group (mean LA volume index, 53.7 ± 20.3 vs. 46.7 ± 13.6, respectively, *P* = .381). Also, the residual potential after the first PV encirclement was slightly higher in the CPAI group than the HPAI group (7.8% vs. 4.7%). The additional ablation for achieving PVI was more frequently performed in the CPAI group compared to the HPAI group. Because the number of total ablation points included the ablation points for achieving the “first PVI”; thus, this resulted in the difference in the number of total ablation points between the two groups. Fifth, although there was no statistically significant difference, at least a numerically larger LA volume index was shown in CPAI than HPAI and CPAI group showed higher variability in the value of LAVI (Figure S6). To provide a supplementary analysis for the difference in LAVI between two groups, we matched 2:1 of the HPAI and CPAI group using similar LAVI (CPAI, n = 26, and HPAI, n = 52). After matching LAVI between two groups, the mean LAVI values of the two groups were not different (47.2 ± 13.6 in the CPAI group and 48.1 ± 13.6 in the HPAI group, *P* = .786, Table S4). In this matched cohort, the HPAI group consistently showed significantly shorter PVI time than the CPAI group (38.6 ± 9.2 vs. 66.1 ± 14.7 minutes, *P* < .001). Thus, higher power ablation with the same target AI could markedly reduce PVI time. Sixth, our strategy for LIPV inferior/posterior lesions was not different in the two groups, and the acute reconnection rates of the two groups were not significantly different (HPAI group vs. CPAI group, 1.2% vs. 3.1%, *P* = .299). We applied 25 W for a 15‐seconds ablation with esophageal temperature monitoring. The acute reconnection rate at LIPV posterior and inferior segments was 1.7%, which was slightly lower, but not significantly iffered from other segments (*P* = .066). Lastly, this study included a small number of patients; thus, the comparison of safety between the two groups might not be conclusive. A high‐power ablation strategy is not a standard technique, and the risk of perforation is an important issue to overcome. We found no complications among 87 patients who received PVI using higher power RF ablation, which would provide significant clinical implications. However, more safety‐related data is needed for higher power strategies to become common.

## 5 CONCLUSION

Higher‐powered ablation can be safely performed using an AI‐guided strategy. Higher‐powered AI‐guided PVI reduced total ablation and total procedure times by a significant reduction in ablation time for PVI, and showed comparable acute PVR rate and long‐term AF‐free survival without significant complications, compared to conventional‐powered AI‐guided PVI.

## CONFLICT OF INTEREST

SRL, HSP, EL, and SO: None; Dr Eue‐Keun Choi received research grants from BMS/Pfizer, Biosense Webster, Chong Kun Dang, Daiichi‐Sankyo, Sanofi‐Aventis, and Skylabs.

## Supporting information

Supplementary Material
