# Supplementary material for: Acute and long‐term efficacy of ablation index‐guided higher power shorter duration ablation in patients with atrial fibrillation: A prospective registry
Source: J Arrhythm. 2021 Jul 21;37(5):1250–9. doi: 10.1002/joa3.12605 (PMC8485805; doi:10.1002/joa3.12605)
Supplement: Supplementary file 1 — Supplementary Material [file JOA3-37-1250-s001.docx]

**Acute and Long-term Efficacy of Ablation Index-Guided Higher Power Shorter Duration Ablation in Patients with Atrial Fibrillation: A Prospective Registry**

**Short title:** Optimal AI-guided higher power PVI

So-Ryoung Lee, MD^1^*, Hyoung-Seob Park, MD, PhD^2^*, Eue-Keun Choi, MD, PhD^1^, Euijae Lee, MD^1^, Seil Oh, MD, PhD^1^

^1^ Department of Internal Medicine, Seoul National University Hospital, Seoul, Republic of Korea

^2^ Division of Cardiology, Department of Internal Medicine, Keimyung University, Dongsan Medical Center, Daegu, Republic of Korea

*Two authors equally contributed.

**Online Supplementary Materials**

**I. Supplementary Tables**

**II. Supplementary Figures**

**I. Supplementary Tables**

**Supplementary Table 1. Details of additional ablation other than PVI**

|  | **HPAI group**  **(n=86)** | **CPAI group**  **(n=32)** | **p-value** |
| --- | --- | --- | --- |
| **PVI only** | 32 (37.2%) | 12 (37.5%) | 0.977 |
| **Additional ablation** | 54 (62.8%) | 20 (62.5%) |  |
| **CTI** | 17 (53.1%) | 48 (55.8%) | 0.994 |
| **LA roof linear** | 8 (25.0%) | 11 (12.8%) | 0.109 |
| **LA posteroinferior linear** | 0 (0%) | 4 (4.7%) | 0.215 |
| **Non-PV trigger** | 4 (12.5%) | 8 (9.3%) | 0.609 |
| **Non-PV trigger, details** | - SVC (n=3)  - Low CT (n=1) | - SVC (n=4)  - Low CT (n=1)  - LA posterior (n=2)  - LA roof (n=2)  - RA upper septum (n=1)  - RA free wall (n=1) |  |
| **Others** | 2 (6.2%) | 2 (2.3%) | 0.295 |
| **Others, details** | - AP (n=2) | - CFAE (n=1)  - TA focal AT (n=1) |  |

Abbreviation: AP, accessory pathway; AT, atrial tachycardia; CFAE, complex fractionated atrial electrogram; CPAI, conventional powered ablation index guided group; CT, crista terminalis; CTI, cavotricuspid isthmus; HPAI, higher powered ablation index guided group; LA, left atrial; PV, pulmonary vein; PVI, pulmonary vein isolation; RA, right atrial; SVC, superior vena cava; TA, tricuspid annulus.

**Supplementary Table 2. Procedural** **data according to segments**

|  | **HPAI group** | **CPAI group** | **p-value** |
| --- | --- | --- | --- |
| **Anterior/roof** | 516 segments | 192 segments |  |
| **Number of ablation points by each segment** | 7.1±3.6 | 8.8±5.7 | <0.001 |
| **Total ablation time for segment (sec)** | 167.8±89.4 | 318.7±209.6 | <0.001 |
| **Mean ablation time by each ablation point (sec)** | 25.9±5.3 | 36.7±5.6 | <0.001 |
| **Mean power (W)** | 39.7±2.4 | 31.3±2.3 | <0.001 |
| **Mean contact force (g)** | 8.9±2.8 | 10.2±3.6 | <0.001 |
| **Mean FTI (g*sec)** | 215.1±44.6 | 349.5±95.5 | <0.001 |
| **Mean AI** | 458.4±17.8 | 460.9±14.6 | 0.079 |
| **Mean impedance drop (ohm)** | 9.8±3.2 | 12.9±4.3 | <0.001 |
| **Posterior/inferior/carina** | 516 segments | 192 segments |  |
| **Number of ablation points by each segment** | 6.4±4.1 | 7.9±4.8 | <0.001 |
| **Total ablation time for segment (sec)** | 116.9±71.6 | 197.5±124.3 | <0.001 |
| **Mean ablation time by each ablation point (sec)** | 17.4±4.0 | 24.1±4.5 | <0.001 |
| **Mean power (W)** | 31.3±3.8 | 25.8±1.6 | <0.001 |
| **Mean contact force (g)** | 9.2±2.5 | 10.8±2.8 | <0.001 |
| **Mean FTI (g*sec)** | 151.0±34.3 | 243.9±47.2 | <0.001 |
| **Mean AI** | 350.6±26.0 | 359.3±19.2 | <0.001 |
| **Mean impedance drop (ohm)** | 8.3±3.3 | 11.2±4.1 | <0.001 |
| **LIPV inferior/posterior** | 172 segments | 64 segments |  |
| **Number of ablation points by each segment** | 5.7±2.8 | 5.5±2.8 | 0.751 |
| **Total ablation time for segment (sec)** | 81.5±37.8 | 91.7±45.2 | 0.119 |
| **Mean ablation time by each ablation point (sec)** | 14.3±2.3 | 15.8±2.3 | <0.001 |
| **Mean power (W)** | 28.0±4.6 | 25.2±1.5 | <0.001 |
| **Mean contact force (g)** | 8.4±2.6 | 8.5±2.4 | 0.794 |
| **Mean FTI (g*sec)** | 116.7±32.6 | 133.1±39.9 | 0.002 |
| **Mean AI** | 292.9±130.1 | 290.5±25.0 | 0.812 |
| **Mean impedance drop (ohm)** | 6.6±2.3 | 9.1±4.0 | <0.001 |

Abbreviation: AI, ablation index; CPAI, conventional-powered AI guided ablation; FTI, force-time integral; HPAI, higher-powered AI guided ablation; LIPV, left inferior pulmonary vein.

**Supplementary Table 3. Comparison of lesion sizes between 40 W and 30 W ablation at the AI 450 in canine models**

|  | **40 W** | **30 W** | **p-value** |
| --- | --- | --- | --- |
| **Right atrium** |  |  |  |
| **Number of ablation points** | 29 | 31 |  |
| **Contact force (g)** | 11.8±1.9 | 11.4±1.8 | 0.295 |
| **Ablation duration (sec)** | 19.8±2.4 | 31.1±2.9 | <0.001 |
| **Force-time integral (g*sec)** | 229±14 | 351±23 | <0.001 |
| **Ablation index** | 462±5 | 457±5 | <0.001 |
| **Impedance drop (ohm)** | 18.5±8.8 | 14.9±11.6 | 0.189 |
| **Maximum lesion width (mm)** | 7.6±0.8 | 7.8±1.8 | 0.761 |
| **Lesion depth** | Transmural | Transmural |  |
| **Steam pop** | 1 (3.4%) | 1 (3.2%) | 0.962 |
| **Right ventricle** |  |  |  |
| **Number of ablation points** | 22 | 20 |  |
| **Contact force (g)** | 12.3±2.0 | 13.4±2.7 | 0.170 |
| **Ablation duration (sec)** | 19.1±1.9 | 29.6±4.1 | <0.001 |
| **Force-time integral (g*sec)** | 232±16 | 383±38 | <0.001 |
| **Ablation index** | 463±5 | 458±4 | 0.001 |
| **Impedance drop (ohm)** | 21.5±10.7 | 20.4±11.6 | 0.762 |
| **Surface lesion width (mm)** | 7.7±1.4 | 8.3±1.5 | 0.135 |
| **Maximum lesion width (mm)** | 8.3±2.1 | 9.1±2.3 | 0.273 |
| **Lesion depth (mm)** | 6.0±1.4 | 5.9±1.9 | 0.841 |
| **Steam pop** | 0 (0%) | 0 (0%) | n/a |

Abbreviation: AI, ablation index.

**Supplementary Table 4. Mean LAVI and procedure related-time among 2:1 matched cohort for LAVI between HPAI and CPAI groups**

|  | CPAI group  (n=26) | HPAI group  (n=52) | p-value |
| --- | --- | --- | --- |
| Mean LAVI (ml/m^2^) | 47.3±13.6 | 48.1±13.6 | 0.786 |
| PVI time (min) | 66.1±14.7 | 38.6±9.2 | <0.001 |
| Total ablation time (min) | 79.1±21.2 | 49.6±15.2 | <0.001 |
| Total procedure time (min) | 198.8±42.4 | 142.5±40.3 | <0.001 |
| Fluoroscopic time (min) | 20.1±7.0 | 14.5±6.4 | <0.001 |

Abbreviation: CPAI, conventional powered ablation index guided group; HPAI, higher powered ablation index guided group; LAVI, left atrial volume index; PVI, pulmonary vein isolation.

**II. Supplementary Figures**

**Supplementary Figure 1. Pulmonary vein segments**

Abbreviation: LIPV, left inferior pulmonary vein; LSPV, left superior pulmonary vein; RIPV, right inferior pulmonary vein; RSPV, right superior pulmonary vein.


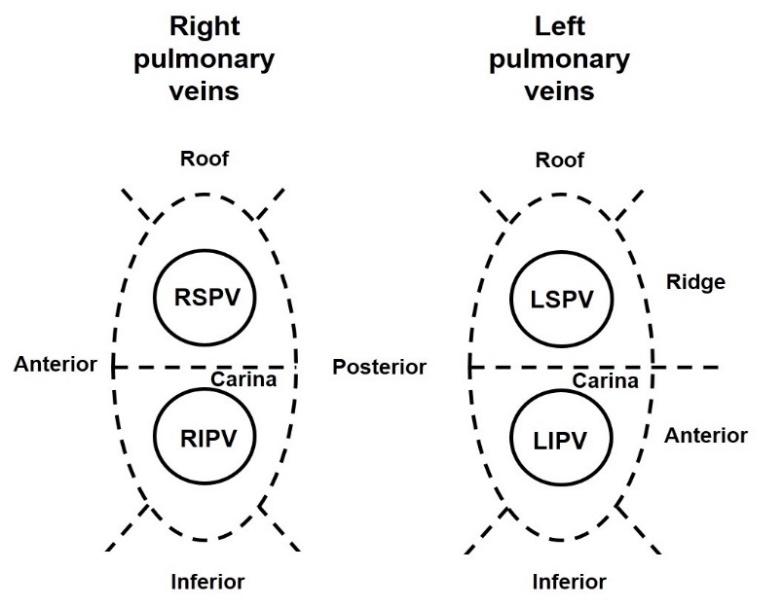


**Supplementary Figure 2. Comparison of ablation parameters between the CPAI group and the HPAI group**


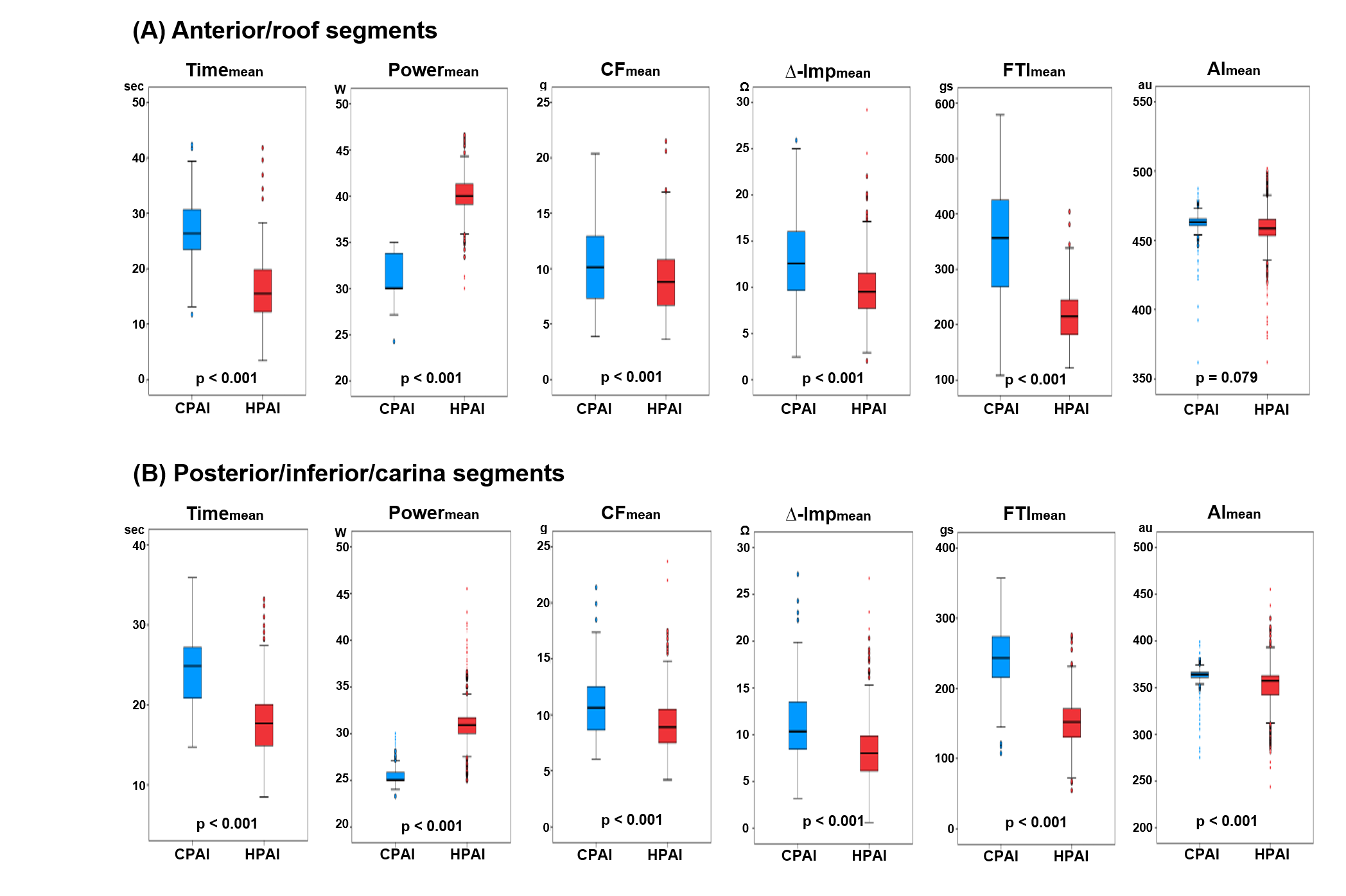


Abbreviations: CF, contact force; FTI, force-time integral; AI, ablation index; Δ-Imp, impedance drop

**Supplementary Figure 3. RP and ER rate of each PV segment in the CPAI and HPAI groups**

**(A) RP rate of each PV segment**

**(B) ER rate of each PV segment**


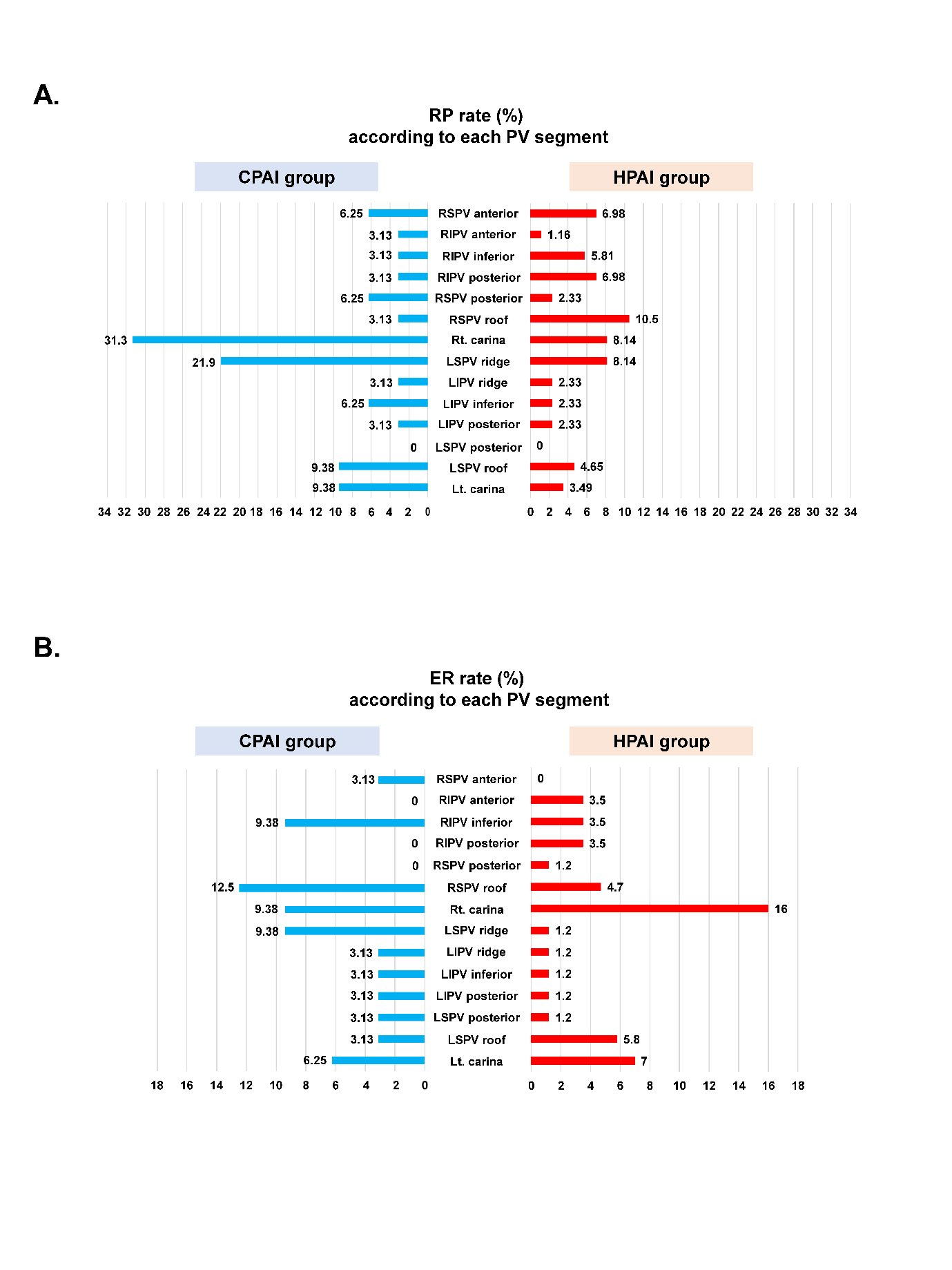
Abbreviations: CPAI, conventional-powered ablation index-guided ablation group; HPAI, higher-powered ablation index-guided ablation group; LIPV, left inferior pulmonary vein; LSPV, left superior pulmonary vein; Lt, left; PV, pulmonary vein; RP, residual potential; RIPV, right inferior pulmonary vein; RSPV, right superior pulmonary vein; Rt, right.

**Supplementary Figure 4. Representative image of lesion sizes at the AI 450 using 30 W and 40 W in canine models**


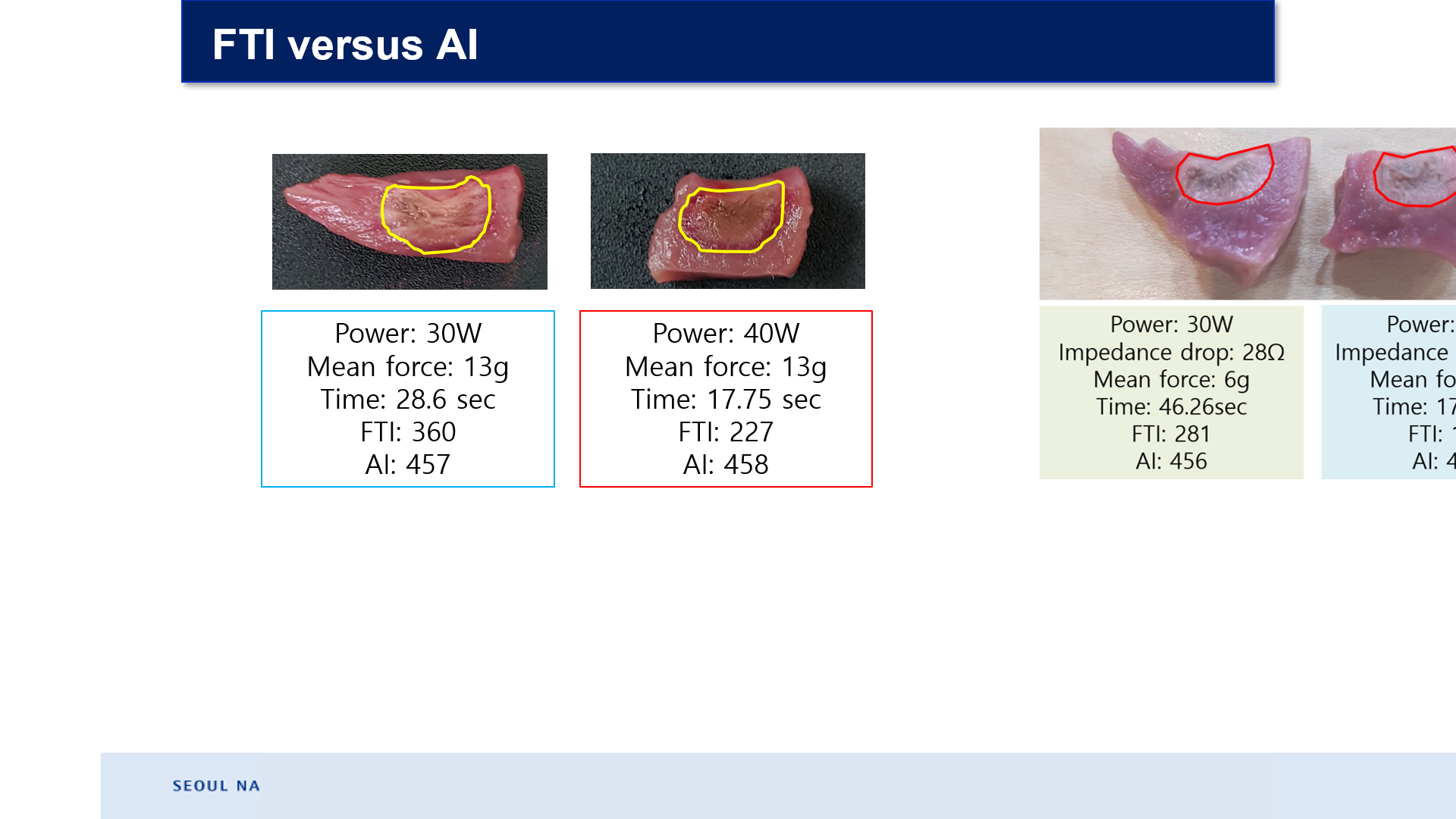


Abbreviations: AI, ablation index; FTI, force-time integral

**Supplementary Figure 5.** **Lesion sizes at the AI 450 using 30 W and 40 W in canine models**


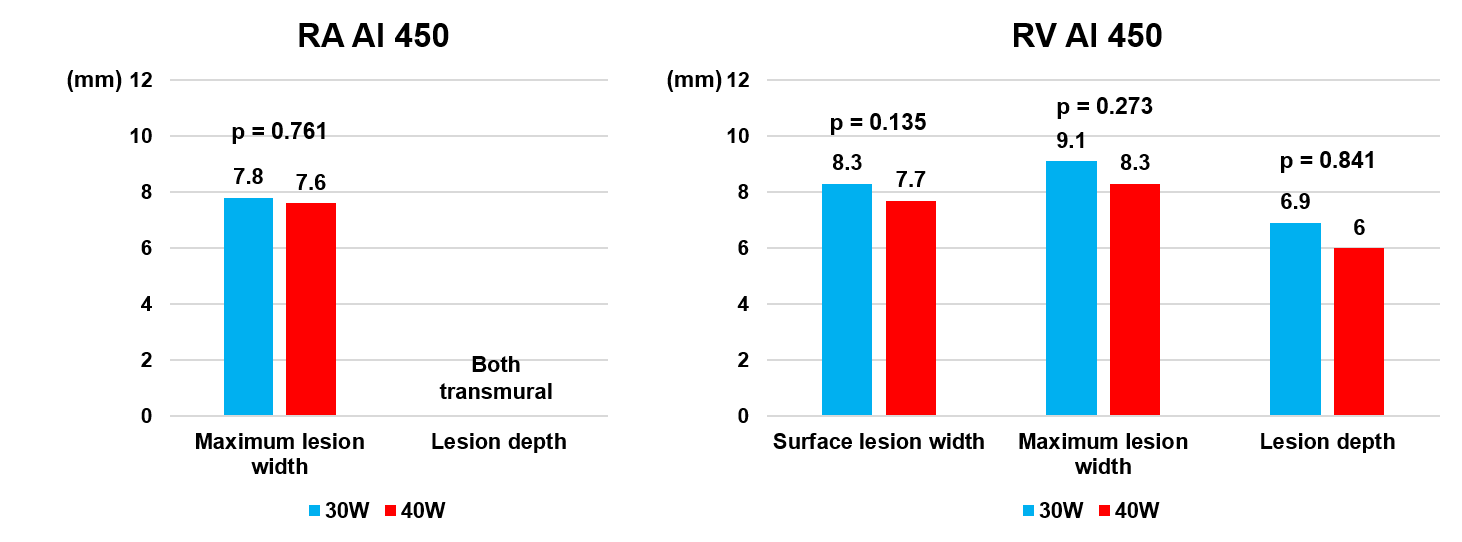


Abbreviation: AI, ablation index; RA, right atrium; RV, right ventricle.

**Supplementary Figure 6. Scatter plot of LA volume index and mean ± 1 standard deviation of each group**


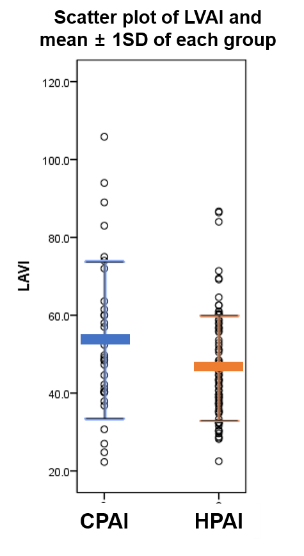


Abbreviation: CPAI, conventional powered ablation index guided group; HPAI, higher powered ablation index guided group; LAVI, left atrial volume index (ml/m^2^).
